# Cholecystokinin in White Sea Bream: Molecular Cloning, Regional Expression, and Immunohistochemical Localization in the Gut after Feeding and Fasting

**DOI:** 10.1371/journal.pone.0052428

**Published:** 2012-12-20

**Authors:** Valeria Micale, Salvatore Campo, Angela D’Ascola, M. Cristina Guerrera, M. Beatrice Levanti, Antonino Germanà, Ugo Muglia

**Affiliations:** 1 Istituto per l’Ambiente Marino Costiero, Consiglio Nazionale delle Ricerche, Messina, Italy; 2 Department of Biochemical, Physiological and Nutritional Sciences, Section of Medical Chemistry, School of Medicine, University of Messina, Messina, Italy; 3 Department of Morphology, Biochemistry, Physiology and Animal Production, Faculty of Veterinary Medicine, University of Messina, Messina, Italy; 4 Consorzio Interuniversitario INBB (Istituto Nazionale Biostrutture e Biosistemi), Roma, Italy; Ecole Normale Supérieure de Lyon, France

## Abstract

**Background:**

The peptide hormone cholecystokinin (CCK), secreted by the midgut, plays a key role in digestive physiology of vertebrates including teleosts, by stimulating pancreatic secretion, gut motility, and gallbladder contraction, as well as by delaying gastric emptying. Moreover, CCK is involved in the regulation of food intake and satiation. Secretion of CCK by the hindgut is controversial, and its biological activity remains to be elucidated. The present paper addresses the regional distribution of intestinal CCK in the white sea bream, *Diplodus sargus*, as well as the possible involvement of hindgut CCK in digestive processes.

**Methodology/Principal Findings:**

Full-lengths mRNAs encoding two CCK isoforms (CCK-1 and CCK-2) were sequenced and phylogenetically analyzed. CCK gene and protein expression levels in the different gut segments were measured 3 h and 72 h after feeding, by quantitative real-time RT-PCR and Western blot, respectively. Moreover, endocrine CCK cells were immunoistochemically detected. Fasting induced a significant decrease in CCK-2 in all intestinal segments, including the hindgut. On the other hand, no significant difference was induced by fasting on hindgut CCK-1.

**Conclusions/Significance:**

The results demonstrated two CCK isoforms in the hindgut of *D.sargus*, one of which (CCK-2) may be involved in the feedback control of uncompleted digestive processes. On the other hand, a functional role alternative to regulation of digestive processes may be inferred for *D.sargus* CCK-1, since its expression was unaffected by feeding or fasting.

## Introduction

The peptide hormone cholecystokinin (CCK), a member from the gastrin-CCK family, has a widespread distribution within the gastro-intestinal tract (GIT), as well as the central and peripheral nervous system in both mammalian and non-mammalian species. In the digestive organs, CCK is synthesized by specific endocrine cells scattered in the intestinal mucosa, as a prepro-CCK polypeptide that is enzymatically cleaved post-translationally to generate biologically active CCK/gastrin-like peptides that share similar carboxy-terminal ends [1]. CCK has been demonstrated to play a key role in digestive physiology of vertebrates including teleosts, by stimulating the release of pancreatic enzymes such as trypsin and chimotrypsin, gut motility, and gallbladder contraction [2], [3], [4], [5], [6] and by delaying gastric emptying [7]. Moreover, it is involved in the regulation of food intake and satiation [8], [9], [10].

CCK mRNA sequences have been determined in several fish species, some of which (i.e., rainbow trout, *Oncorhynchus mykiss* [11], Japanese flounder, *Paralichthys olivaceus* [12], green pufferfish, *Tetraodon nigroviridis* [12], and Atlantic salmon, *Salmo salar* [13], displayed different CCK isoforms, belonging to two major phylogenetic clusters, i.e. CCK1 and CCK2.

Immunohistochemical studies conducted in a number of fish larvae, such as the sea bass, *Dicentrarchus labrax* [14], turbot, *Scophthalmus maximus* [15], Japanese flounder, *Paralichthys olivaceus* [16], Atlantic halibut, *Hippoglossus hippoglossus* [17], bluefin tuna, *Thunnus thynnus* [18], ayu, *Plecoglossus altivelis* [19], red drum *Sciaenops ocellatus (L.)* [20], have demonstrated the presence of CCK-immunoreactive cells (CCK-ir) in the midgut, and a possible functional relationship has been suggested between the macroscopic anatomy of the larval digestive tract and the distribution pattern of CCK-producing cells, with CCK cells concentrated in the anterior midgut and pyloric caeca (where the ingested food is retained longest and can be best attacked by digestive enzymes) in species with a rotated gut, and, conversely, scattered throughout the whole midgut in species with a straight gut (ayu, herring) [21]. All the above cited studies failed to demonstrate the occurrence of CCK-producing cells in the larval hindgut. Only recently, the occasional presence of CCK-ir cells has been reported in the hindgut of Atlantic cod larvae [22]. Moreover, our recent study on the ontogeny of CCK-ir cells in larval sharpsnout seabream, *D. puntazzo* [23], demonstrated the occurrence of CCK-ir cells in the hindgut of all examined larvae, in a proportion varying between 16% and 30% of total intestinal CCK-ir cells.

In adult fish, CCK in the hindgut has been demonstrated by means of RT-PCR or immunohistochemistry in four species (rainbow trout, *O.mykiss* [11], Japanese yellowtail, *Seriola quinqueradiata* [24], turbot, *S. maximus (L.)* [25], and Atlantic salmon, *S. salar* [13], while it has been excluded in five (gilthead sea bream, *Sparus aurata* [26], [27], Japanese flounder, *P. olivaceus* [28], Korean aucha perch, *Coreoperca herzi* [29], brown trout, *Salmo trutta,* [30]and red drum, *S. ocellatus* [20].It has been hypothesized that hindgut CCK-ir cells might participate in the feedback control of digestive processes, by receiving chemical signals from uncompletely digested food reaching the hindgut [22]. However, the finding of different expression patterns of multiple intestinal CCK isoforms in the same individual, as in both rainbow trout and Atlantic salmon [11], [13], is also suggestive of different roles exerted by midgut and hindgut CCK.

In the present paper, the sequences of full-length mRNAs encoding CCK/gastrin-like peptide hormones were identified and phylogenetically analyzed for the first time in the white sea bream, *Diplodus sargus* (L.) a teleost fish (Sparidae, Perciformes) candidate for diversification in Mediterranean aquaculture [31], [32]. Moreover, the distribution of CCK-ir cells, as well as CCK gene and protein expression in the different gut segments were evaluated in fed and fasted fish, by a combined molecular and immunological approach, in order to assess a possible relationship between hindgut CCK and digestive processes.

## Materials and Methods

### Ethics Statement

The experimental protocol was in accordance with the principle outlined in the Declaration of Helsinki and with the National law regarding the care and use of laboratory animals (National Law n. 116/1992). The Fish Care Committee of C.I.S.S., University of Messina, Italy, specifically approved this study. Rearing, handling and killing procedures were approved by the Fish Care Committee of C.I.S.S., University of Messina, Italy.

### Animals and Sampling

**Figure 1:**
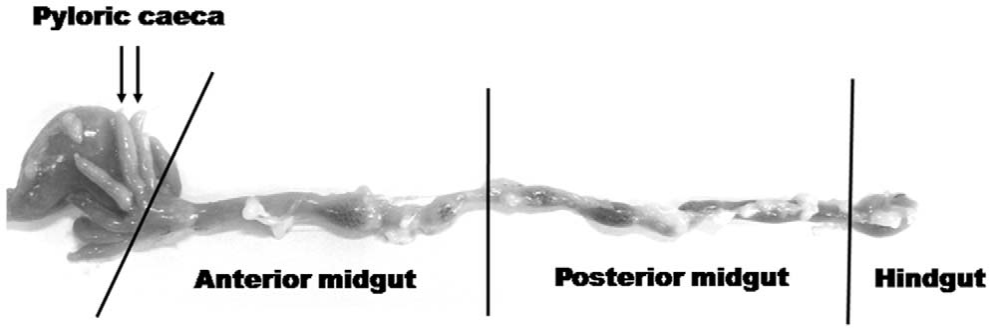
Extracted digestive tract from an adult white sea bream, *D. sargus*. Intestinal segments sampled for immunohistochemistry, Western blot, and qPCR are indicated on the figure.

White sea bream, *Diplodus sargus* L., in the male phase(mean body weight 93.5±7.1 g), were reared at the C.I.S.S. (Experimental Ichthyopathology Centre of Sicily, Veterinary Faculty, University of Messina, Italy) in 300 l indoor tanks which were part of a recirculating seawater system (T = 19°C). The fish were fed a commercial pellet (ALLER AQUA, Christiansfeld, Denmark), administered continuously by an automatic feeder. Prior to the fasting experiments, five fish were sampled for cDNA cloning (see below). The regional distribution of CCK-IR cells, CCK mRNA expression, and CCK protein expression in the gut, before and after starvation, was evaluated by immunohistochemistry, quantitative real-time RT-PCR (qPCR) and quantitative Western blot, respectively. For this purpose, ten fish (fed group) were sampled three hours after stopping the feeder, and ten (starved group) 72 h later. Fish were euthanized by an overdose of MS222 and the gut was rapidly removed and dissected into four segments (pyloric caeca, anterior midgut, posterior midgut, and hindgut) (Fig. 1). For qPCR and Western blot (*n* = 6+6), the dissected segments were immediately immersed in liquid nitrogen and stored at −80°C until RNA extraction was performed. For immunohistochemistry (*n* = 4+4), the dissected segments were fixed in Bouin's solution, dehydrated through graded alcohols, cleared in xylene, and embedded in paraffin.

### Molecular Cloning of White Sea Bream cDNA Cholecystokinins

Total RNA was extracted from white sea bream whole gut using Trizol reagent kit (Invitrogen, USA) according to manufacturer’s instructions. Total RNA were processed by 5′ and 3′ Gene Rapid Amplification of cDNA ends method (RACE) [33], [34], using 5′ and 3′ Gene Racer kits (Invitrogen, USA). To obtain forward and reverse Gene Specific Primers (GSP, Table 1) we separately aligned the CCK-1 and CCK-2 mRNAs nucleotide sequences from four teleosts, i.e. *Paralichthys olivaceus* (GenBank Accession Numbers: AB009281 and AB086399), *Tetraodon nigroviridis* (GenBank Accession Numbers: AB086401 and AB086402), *Seriola quinqueradiata* CCK-1 (GenBank Accession Number: AB205406) and *Clupea harengus* CCK-2 (GenBank accession number: AY334083). Nucleotide sequences were aligned by CLUSTAL-W software, whereas the high homology regions were used to obtain the GSPs shown in Table 1; the same alignment was performed for β-actins, in order to find forward and reverse primers for white sea bream partial β-actin mRNA sequence identification. Reverse transcriptions were performed using the Superscript III kit (Invitrogen, USA). The cDNAs for 5′-RACE were obtained with the Gene Specific CCK-1, CCK-2 3_Reverse primers, while the cDNAs for 3′-RACE were synthesized using the poly(T)-anchor primer, included in the kit. The β-actin amplicon was directly produced by using Superscript III One-Step RT-PCR Platinum Taq HiFi kit (Invitrogen, USA), following instructions provided by the manufacturer. All cDNAs synthesized were used to perform two 5′-RACE and two 3′-RACE PCR for white sea bream CCK-1 and CCK-2. For 5′-RACE, the target cDNA was amplified using an appropriate amount of the AAP (Abridge Danchor Primer) gene specific and the CCK-1, or CCK-2 2_Reverse primer. A second (nested) PCR was performed using the Abridged Universal Amplification Primer (AUAP) and the second gene specific CCK-1, or CCK-2 1_Reverse primer, as above described. The PCR products were visualized in 2% HR agarose gel (EuroClone, UK) by fluorochromatizaton with ethidium bromide. The detected amplicons were excised from the gel and purified on a JETquick spin column kit (Genomed, USA), as suggested by the manufacturer. For 3′-RACE the first strand cDNA synthesized by the poly(T)-anchor primer was purified and subjected to a first PCR reaction by using an appropriate amount of the AUAP and the CCK-1, or CCK-2 gene specific 2_Sense primer. A nested PCR was then performed, by using the same amount of AUAP and the CCK-1, or CCK-2 second gene specific 1_Sense primer. The amplicons were visualized and purified as above. The purified amplicons were cloned by the TOPO TA Cloning sequencing kit (Invitrogen, USA). Clones were screened by nucleotide sequencing with both 5′(T3) and 3′(T7) oligos and the BigDye terminators cycle sequencing kit v. 1.1 (Applied Biosystems, USA), as suggested by the manufacturer. The obtained polynucleotide fragments were separated by capillary electrophoresis on a mod. 310 Genetic Analyzer (Applied Biosystems, USA). For each cholecystokinin, the 5′end and the 3′ end nucleotide sequences identified were manually overlapped in order to obtain the complete mRNA sequence. The detected nucleotide sequences were analyzed by the on line ExPasy translate tool (http://web.expasy.org/translate/) in order to obtain the predicted proteins.

**Table 1:** Name and nucleotide sequence of primers used for white sea bream CCK-1, CCK-2, and β-actin nucleotide sequence identification.

NAME	GENE SPECIFIC PRIMERS
CCK-1 1_Reverse 5′-RACE	5′- GTACACAGGACTGCCAGCAC-3′
CCK-1 2_Reverse 5′-RACE	5′- GACTGCCAGCACGACACA-3′
CCK-1 3_Reverse 5′-RACE	5′-ACAAATGTACAACAAATACATCATAAATAG-3′
CCK-1 1_Sense 3′-RACE	5′-CTATTTATGATGTATTTGTTGTACATTTGT-3′
CCK-1 2_Sense 3′-RACE	5′-TGTGTCGTGCTGGCAGTC-3′
CCK-2 1_Reverse 5′-RACE	5′-TCTGTCCTTTATCCTGTGGC-3′
CCK-2 2_Reverse 5′-RACE	5′-CTGTGGCCCGGGGCAAGA-3′
CCK-2 3_Reverse 5′-RACE	5′-CAAAGTCCATCCAGCCGA-3′
CCK-2 1_Sense 3′-RACE	5′-AACAGCCTGAACCAGCTT-3′
CCK-2 2_Sense 3′-RACE	5′-TCTCCAGGAAAGGCTCTCC-3′
β-actin 1_Sense (Direct Amplification)	5′-GGAGAAGATCTGGCATCACA-3′
β-actin 1_Reverse (Direct Amplification)	5′-CCTCCGATCCAGACAGAGTATT-3′

### Sequence Analysis and Phylogenetic Tree Computation

The deduced white sea bream cholecystokinin amino acid sequences were aligned with those of other teleosts using the CLUSTAL-W tool enclosed in the MEGA 5.05 free ware software [35](http://www.megasoftware.net), whereas *Homo sapiens*, *Strutio camelus*, *Python molurus* and *Xenopus laevis* CCKs were included in the study as an outgroup (Table 2). After manual correction, the alignments were computed by means of the online ProTest program (http://darwin.uvigo.es/software/prottest_server.html) in order to assess the best-fit matrix for understanding CCK-1 and CCK-2 evolution: the JTT model [36]was chosen as transition probability matrices for constructing the CCKs (31 Taxa) tree and was calculated as distance of amino acid substitution per site. The phylogenetic tree was estimated by three different methods: the tree topology was assayed by the Neighbor-Joining method [37]of the MEGA v. 5.05 program, with 1000 replicates for the bootstrap test; the phylogenetic tree was evaluated by the Maximum Likelihood (ML) method implemented in the PHYML program [38](http://www.abc.se/~nylander/), as well as by a newly developed Bayesian inference method included in the MrBayes v.3.1.2 package [39](http://mrbayes.net). We estimated by PHYML program robustness non parametric bootstrap analysis with 500 replicates; the proportion of invariable sites was evaluated during the analysis, whereas the range of rate variation across site was determined by gamma distribution set to 5 number of categories. A Bayesian Markov Chain Monte Carlo (MCMC) analysis was also performed by MrBayes software, for testing evolutionary hypotheses in which the tree was weighed proportionally to its posterior probability. For this calculation, the JTT model was chosen as a substitution matrix, the site of heterogeneity was gamma with 4 number of categories, while the number MCMC replicates was 10^6^. The final results were summarized in a best tree, after discarding the first 25% obtained samples. Finally, the phylogenetic tree was viewed by using the free ware FigTree software.

**Table 2:** Taxa, species, and CCK protein sequences investigated in the present study.

Taxon and Species	Proteins	AccessionNumber/Reference
Outgroup		
***Homo sapiens***	CCK	CAG47022
***Struthio camelus***	CCK	CAB62255
***Python molurus***	CCK	AAM77662
***Xenopus laevis***	CCK	CAA87639
***Leucoraja ocellata***	CCK	ACH42756
***Squalus acanthias***	CCK	CAB94727
**Teleosts**		
*Anguilla japonica*	CCK	BAD01500
*Paralichthys olivaceus*	CCK-1	BAA23734
	CCK-2	BAC44892
*Seriola quinqueradiata*	CCK	BAE16613
*Salmo salar*	CCK	ACM09682
*Pseudopleuronectes* *americanus*	CCK	ACH42757
*Oncorhynchus mykiss*	CCK-1	NP_001117817
	CCK-L	CAA09907
	CCK-T	CAA09906
	CCK-N	NP_001118083
*Ictalurus punctatus*	CCK	BE212760
*Tetraodon nigroviridis*	CCK-1	BAC44894
	CCK-2	BAC44895
*Clupea harengus*	CCK	AAQ17201
*Gadus morhua*	CCK	ADG64736
*Danio rerio*	CCK-V	XP_002665661
	CCK	XP_001346140
*Sciaenops ocellatus*	CCK	ACF04738
*Carassius auratus*	CCK	O93464
*Oryzias latipes*	CCK-1	xd27fq/ENSORLP00000007034*
	**CCK-2**	37pxvj/ENSORLP00000007487*
*Takifugu rubripes*	CCK-1	jm6771/ENSTRUP00000034252**°**
	**CCK-2**	tdz3j7/ENSTRUP00000004505**°**

* Ensembl Medaka ver. 50.1f.

°Ensembl Fugu ver. 50.4j.

### Regional Analysis of CCK Expression and CCK-ir Cell Distribution in the Gut after Feeding and Fasting

#### 1. Quantitative real-time RT-PCR (qPCR)

A treatment with DNase I (Fermentas Inc., Canada) was applied to total RNA to prevent genomic DNA contamination. Purified total RNA was used to synthesize the first strand of cDNA by High-capacity c-DNA Archive Kit (Applied Biosytem, USA). Quantitative real-time RT-PCR was performedby using TaqMan probes and primers ready to use (Assays on Demand, Applied Biosystems, USA), the Universal TaqMan PCR Master Mix and the mod. 7500 PCR Real Time System instrument (Applied Biosystems, USA). β-actin mRNA was used as endogenous control to allow the relative quantification of CCK-1 and CCK-2. The sequence of *Diplodus sargus* mRNA β-actin was previously characterized and submitted to the GenBank database (accession number JN210581). Primers and probes nucleotide sequences were designed by Primer Express program (Applied Biosystems, USA) as shown in Table 3. PCR products were quantified by measuring thresholds cycle (Ct) of targets and endogenous control. The results were calculated through the 2^−ΔΔCt^ algorithm against β-actin, and expressed as the n-fold difference compared to an arbitrary calibrator, chosen as a higher value than ΔΔCts obtained. All assays were carried out in triplicate. CCK mRNA levels in the different intestinal segments were compared by one-way analysis of variance (ANOVA), followed by Tukey’s test. Results of the effect of starvation were analyzed using Student’s *t*-test. Differences between groups were considered to be significant if *p*≤0.05.

**Table 3:** Name and nucleotide sequence of primers and probes used for mRNA expression analysis of white sea bream CCK-1, CCK-2, and β-actin by qPCR.

NAME	PRIMERS AND PROBES SEQUENCES
CCK-1 Sense REAL TIME	5′- ACCACAGGGATAACTGGCTTGT-3′
CCK-1 Reverse REAL TIME	5′- CCGACATCGAAGGATCAAAAA-3′
CCK-1 Probe REAL TIME	5′- AGCGTTCATAGCGACGTC-3′
CCK-2 Sense REAL TIME	5′- CAGGAAAGGCTCTCCCTACCA-3′
CCK-2 Reverse REAL TIME	5′- TCCATCCAGCCGAGGTAGTC-3′
CCK-2 Probe REAL TIME	5′- CCCATGGATAAAGGAC -3′
β-actinSense REAL TIME	5′- GCACCCTGTCCTGCTCACA -3′
β-actinReverse REAL TIME	5′-GTTGAAGGTCTCGAACATGATCTG-3′
β-actinProbe REAL TIME	5′- CCAACAGGGAGAAGATGA -3′

#### 2. Quantitative western blot analysis

For SDS-PAGE and Western blotting, intestinal tissue samples were washed twice in ice-cold PBS and subsequently homogenized in 1 mL lysis buffer (20 mM HEPES, pH 7.6, 1.0 mM dithiothreitol, 1.0 mM EGTA, 1% Triton, 50 mM β-glycerol phosphate, 10% glycerol, 0.5 mM phenyl methylsulphonyl fluoride, aprotinin, leupeptin, pepstatin A (10 µg·mL^−1^ each) and 100 mM Na_3_VO_4_. After protein determination using the Bio-Rad protein assay kit (Bio-Rad, Richmond, CA, USA), aliquots of whole cell protein extract (30 µg/well) were denatured and separated by electrophoresis on 12% SDS polyacrylamide gel. The proteins were blotted onto polyvinylidene difluoride membranes (Amersham Biosciences) using a semidry apparatus (Bio-Rad). The membranes were then incubated overnight in a roller bottle with the specific diluted (1∶500) primary antibody (anti-flounder CCK-10 antiserum raised in rabbit, [16]) in 5% non-fat dry milk, 1×TBS, and 0.1% Tween 20 at 4°C. After washing three times in a washing buffer (1×TBS, 0.1% Tween 20), the blots were incubated 1 h at room temperature with the diluted (1∶20000) polyclonal antibody (goat anti-rabbit conjugated with peroxidase), in 1×TBS containing 0.15 Tween-20 and 5% non-fat dry milk and the proteins were analysed by the enhanced chemiluminescence system, according to the manufacturer's protocol (Chemicon International, California, USA). The protein signal was quantified by scanning densitometry using a bio-image analysis system (Bio-Profil Celbio, Milan, Italy). β-actin was used as endogenous control, by incubating the membrane overnight at 4°C with a primary antibody mAb, Mouse (GenScript USA, Inc.), at a dilution of 1∶1000, followed by washing and incubation with a secondary antibody (goat anti-mouse conjugated with peroxidase, 1∶20000) 1 h at room temperature.Results were expressed as quantitative relative amounts. CCK protein levels in the different intestinal segments were compared by one-way analysis of variance (ANOVA), followed by Tukey’s test. Results of the effect of starvation were analyzed using Student’s *t*-test. Differences between groups were considered to be significant if *p*≤0.05.

#### 3. Immunohistochemical localization of CCK cells in the gut

10 µm thick randomly selected sagittal sections were cut from paraffin embedded tissue samples, and collected on gelatin-coated microscope slides. The sections were processed for indirect peroxidase immunohistochemistry as described elsewhere [23]. Briefly, deparaffinized and rehydrated sections were rinsed in Tris/HCl buffer (0.05 M, pH 7.5) containing 0.1% bovine serum albumin and 0.2% Triton X 100. Endogenous peroxidase activity was blocked by 3% H2O2, and sections were incubated overnight at 4°C with the primary anti-flounder CCK10 antiserum raised in rabbit [16], diluted 1∶100 in Trizma/HCl Buffered Saline (TBS). After incubation, sections were washed and incubated for 90 min at room temperature with peroxidase-labelled sheep anti rabbit IgG (Amersham, Bucks., UK), diluted 1∶100 in TBS, and the immunoreaction was visualized using 12.5 µg of 3–3′diaminobenzidine diluted in 25 ml of TBS with 37.5 µl of H_2_O_2_, as chromogen. Control sections were processed in the same way, substituting the primary antibody by a non-immune rabbit serum or omitting the primary or the secondary antibodies in the incubation.

## Results

### Structure, Comparison of Amino Acid Sequences, and Phylogenetic Analysis of White Sea Bream Cholecystokinins

**Figure 2:**
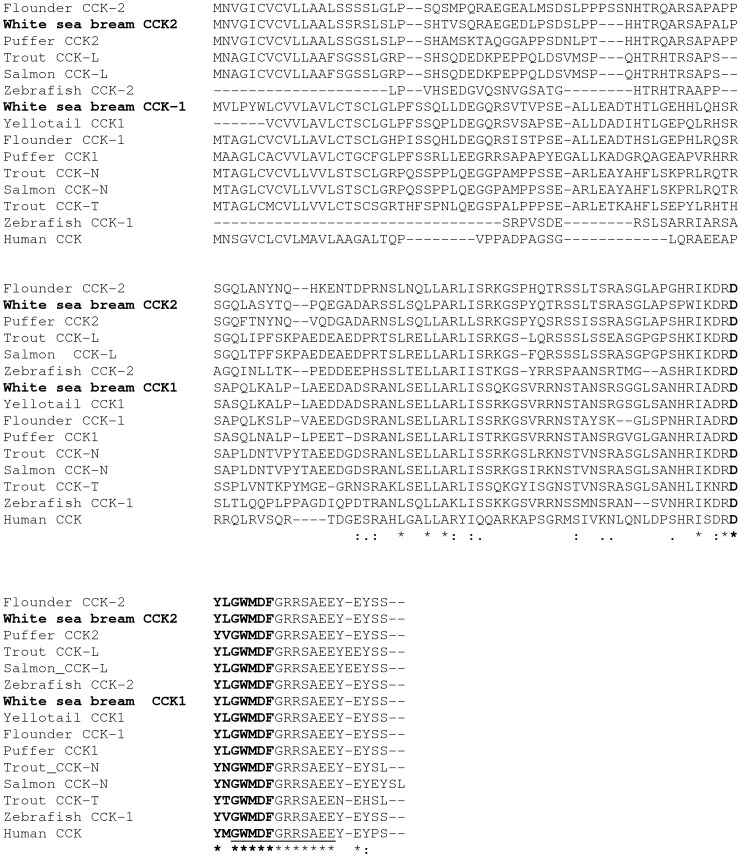
Alignment of CCK amino acid sequences from various teleost fish and human by CLUSTAL-W tool. Consensus key: (*) single, fully conserved residue; (:) conservation of strong groups; (.) conservation of weak groups; () no consensus. The octapeptide is in bold characters. GenBank Accession Nos. are as reported in Table 2.

Two different full-length nucleotide sequences were obtained for white seabream CCK mRNAs, together with a partial sequence of β-actin, which have been deposited in GenBank. The white seabream CCK-1 mRNA (Genbank Accession No. JN210578) is 805 bp in length up to the poly(A) site, which contains a 414 bp open reading frame (ORF) encoding a putative protein of 137 amino acids. The ATG start codon begins at position 71, the TAA stop codon ends at position 484 and the poly(A) signal is located at position 787. The predicted prepro-CCK1 peptide includes a C-terminal octapeptide, DYLGWMDF. The white sea bream CCK-2 mRNA (Genbank Accession No. JN210579) is 811 bp in length up to the poly(A) site which contains a 399 bp ORF, encoding a putative protein of 132 amino acids. The ATG start codon is located at position 75, the TAA stop codon at position 473 and the two poly(A) signals at position 556 and 764, respectively. The C-terminal octapeptide of the predicted prepro-CCK2 peptide is the same as CCK-1, DYLGWMDF. The white seabream CCK-1 and CCK-2 amino acid sequences were aligned with those of other teleost and human (Fig. 2).White seabream CCK-1 displayed a high degree of homology (84%) with both flounder CCK1 and yellowtail, followed by puffer CCK1 (76%), trout and Atlantic salmon CCK-N (68%), and both zebrafish CCKs (61%), whereas white seabream CCK-2 displayed a high degree of homology (80%) with both flounder and puffer CCK2, followed by trout and Atlantic salmon CCK-L (62–63%), and zebrafish CCKs (53–57%). Lower homology (40–50%) occurred with the other aligned sequences, including the human, except for the putative CCK-8 carboxyterminal portion, that was identical in all species, except for the third amino acid of the octapeptide sequence.

**Figure 3:**
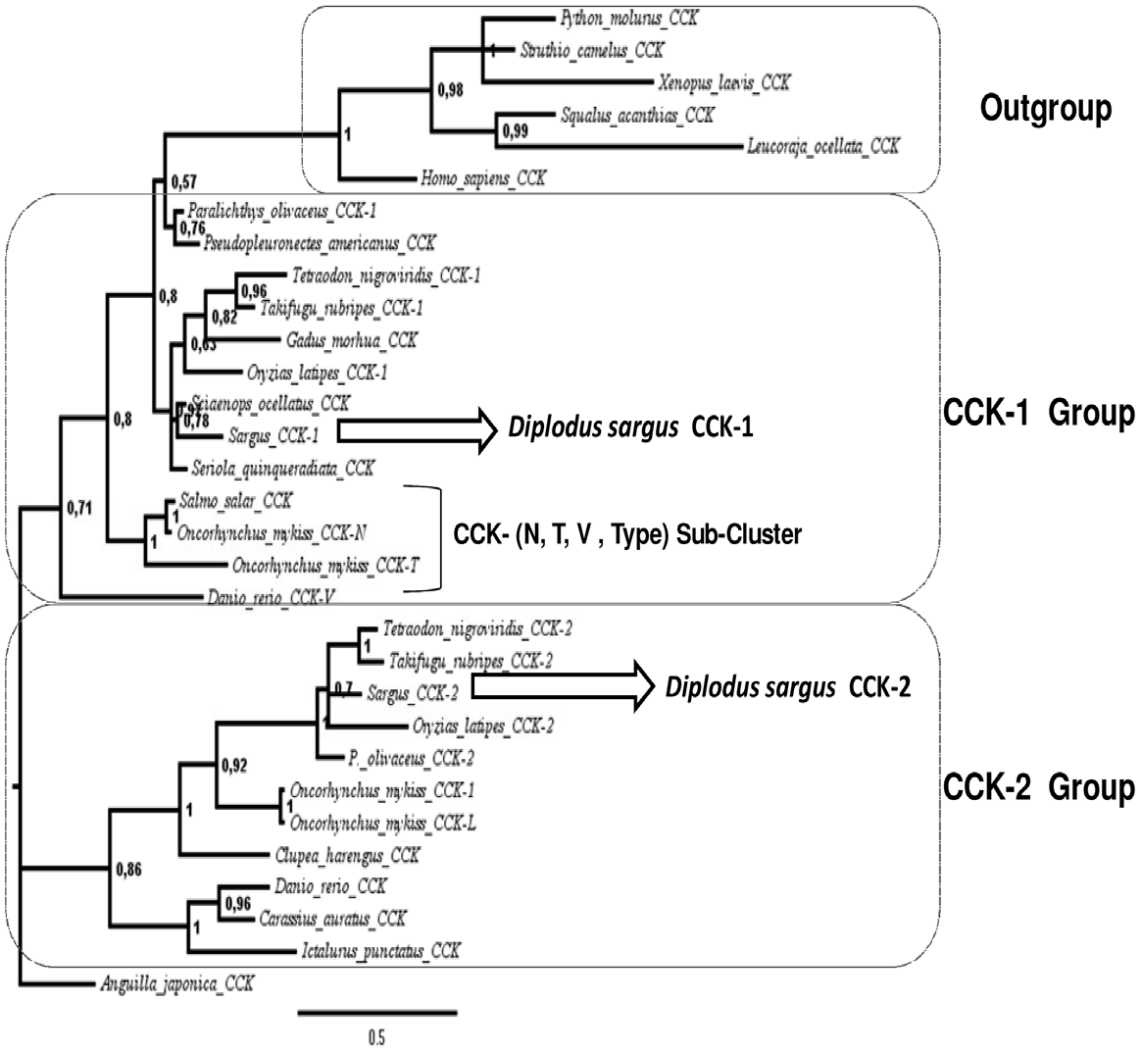
Phylogenetic analysis of CCK protein sequences from *D. sargus* and various species. The best phylogenetic tree was obtained by Bayesian Markov Chain Monte Carlo (MCMC) method, using the JTT substitution model for 10^6^ replicates, after discarding the first 25% obtained samples. The scale bar indicates the substitution rate per site. Numbers at nodes indicate posterior probability (range 0–1). Protein IDs (GenBank Accession Nos) are as reported in Table 2.

A phylogenetic analysis of the inferred white sea bream CCK-1 and CCK-2 protein sequence and CCK sequences from various vertebrates, including teleosts (Fig. 3) was performed. The data set was used to calculate the best CCK tree, using the neighbor-joining algorithm, the Maximum Likelihood (ML) approach and the Bayesian MCMC method, the latter used for the first time to analyze CCK phylogenetic evolution. The tree topology reported in this study is congruent with the neighbor-joining assay (data not shown). The trees of CCKs built by Maximum Likelihood and Bayesian inference algorithms were quite similar (data not shown), although the Bayesian tree was better worked out. In fact, Bayesian CCK tree appears very well resolved with values at the nodes upper 0.5. The phylogenetic tree divided the CCK family into two major groups, namely the teleostean CCK-1 and CCK-2 subfamilies, to which white sea bream CCK-1 and CCK-2, respectively, belong, as well as an outgroup, originating from a branch of the CCK-1 group, including tetrapods, strongly clustered with cartilagineous fish. A sub-cluster including Atlantic salmon CCK, rainbow trout CCK-T and CCK-N, and zebrafish CCK-V was derived from another branch of the same CCK-1 group, while eel CCK originated independently from the analyzed species. White sea bream CCK-1 is positioned in a homophyletic way with *Sciaenops ocellatus* CCK, whereas white sea bream CCK-2 shares a homophyletic position with *Oryzias latipes* CCK-2.

### Effect of Feeding and Fasting on Intestinal CCK

#### 1. CCK mRNA expression (Fig. 4)

Regional analysis of CCK-1 and CCK-2 mRNA expression along the intestinal tract was performed by qPCR 3 and 72 hours after feeding. CCK-2 was most strongly expressed in pyloric caeca and anterior midgut in both fed and starved fish, whereas a significantly lower expression was found in posterior midgut and hindgut. Fasting induced a significant decrease in CCK-2 in all intestinal segments. CCK-1 was expressed in variable amounts in all intestinal segments, regardless of intestinal topography. Fasted fish showed significantly higher levels of CCK-1 in pyloric caeca, anterior and posterior midgut, compared to fed fish. On the other hand, no significant difference was induced by fasting on hindgut CCK-1 (P≤0.05).

**Figure 4:**
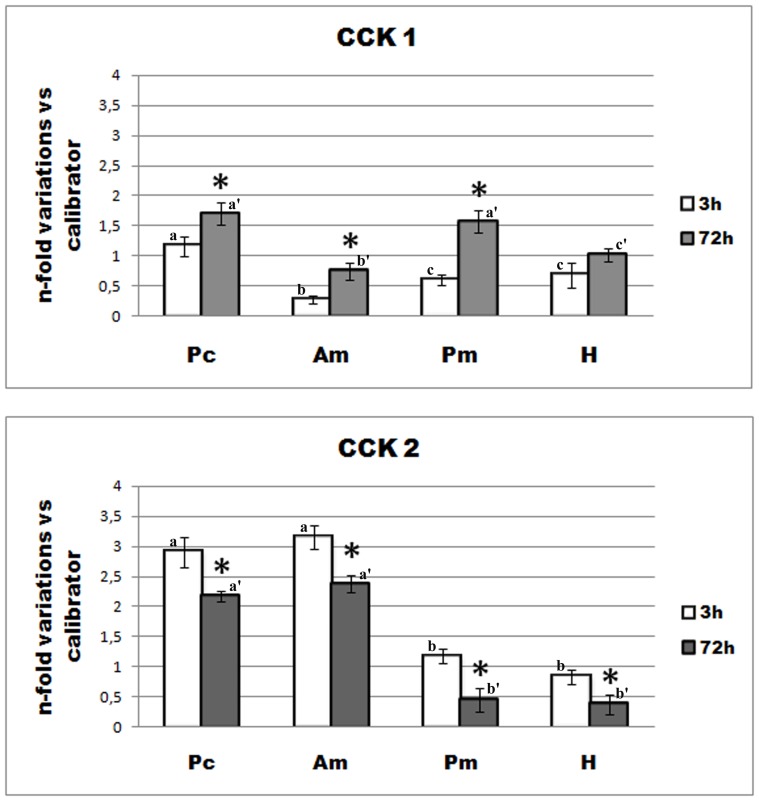
Effect of feeding and fasting on regional expression of *D.sargus* CCK-1 and CCK-2 mRNA. CCK-1 and CCK-2 mRNA levels were measured by real-time quantitative RT-PCR (qPCR) *vs* an arbitrary calibrator in the different intestinal segments of fed (white bars) and fasted (grey bars) white sea bream. Bars with different letters are significantly different (*p*≤0.05). Error bars represent standard error of the mean (*n* = 6 fish). *p≤0.05, significant differences between sampling times. Pc: pyloric caeca; Am: anterior midgut; Pm: posterior midgut; H: hindgut.

#### 2. CCK protein expression (Fig. 5)

Quantitative analysis of CCK protein expression by Western blot revealed a significant decrease induced by 3 days starvation in all intestinal segments, except the hindgut, where no significant difference occurred.

**Figure 5:**
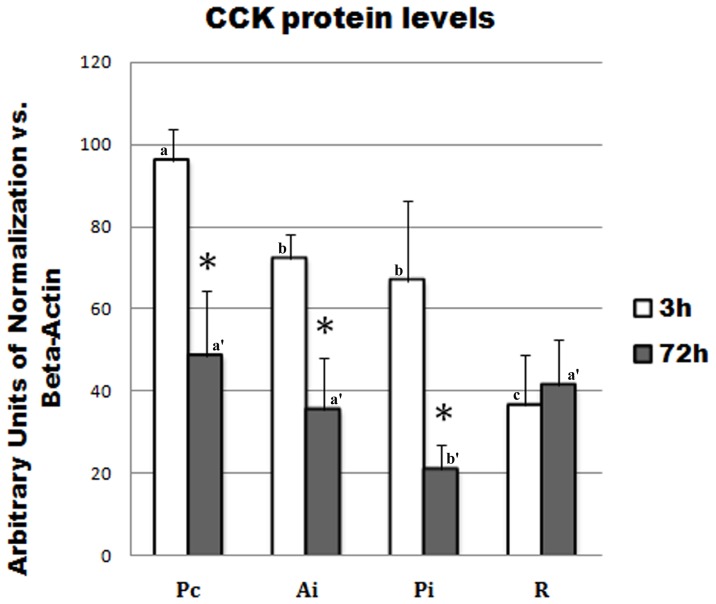
Effect of feeding and fasting on regional expression of *D.sargus* CCK protein. CCK protein levels were measured by quantitative Western blot *vs* β-actin in the different intestinal segments of fed (white bars) and fasted (grey bars) white sea bream. Bars with different letters are significantly different (*p*≤0.05). Error bars represent standard error of the mean (*n* = 6 fish). *p≤0.05, significant differences between sampling times. Pc: pyloric caeca; Am: anterior midgut; Pm: posterior midgut; H: hindgut.

#### 3. CCK-ir cell distribution (Fig. 6)

All examined samples from both fed and fasted fish displayed CCK immunoreactive cells (CCK-ir) in each considered segment. CCK-ir cells appeared variable in shape (triangular, spindle-shaped, ovoid or circular), according to sectioning. When triangular, they showed an apex pointing towards the lumen and a basal part, mainly located in the mucosal lamina propria. CCK-ir cells were mainly concentrated in pyloric caeca, and gradually decreased along the midgut and hindgut.Fasting appeared to induce a pronounced decrease in CCK-ir cell density in the pyloric caeca, anterior and posterior midgut, but not in the hindgut.

**Figure 6:**
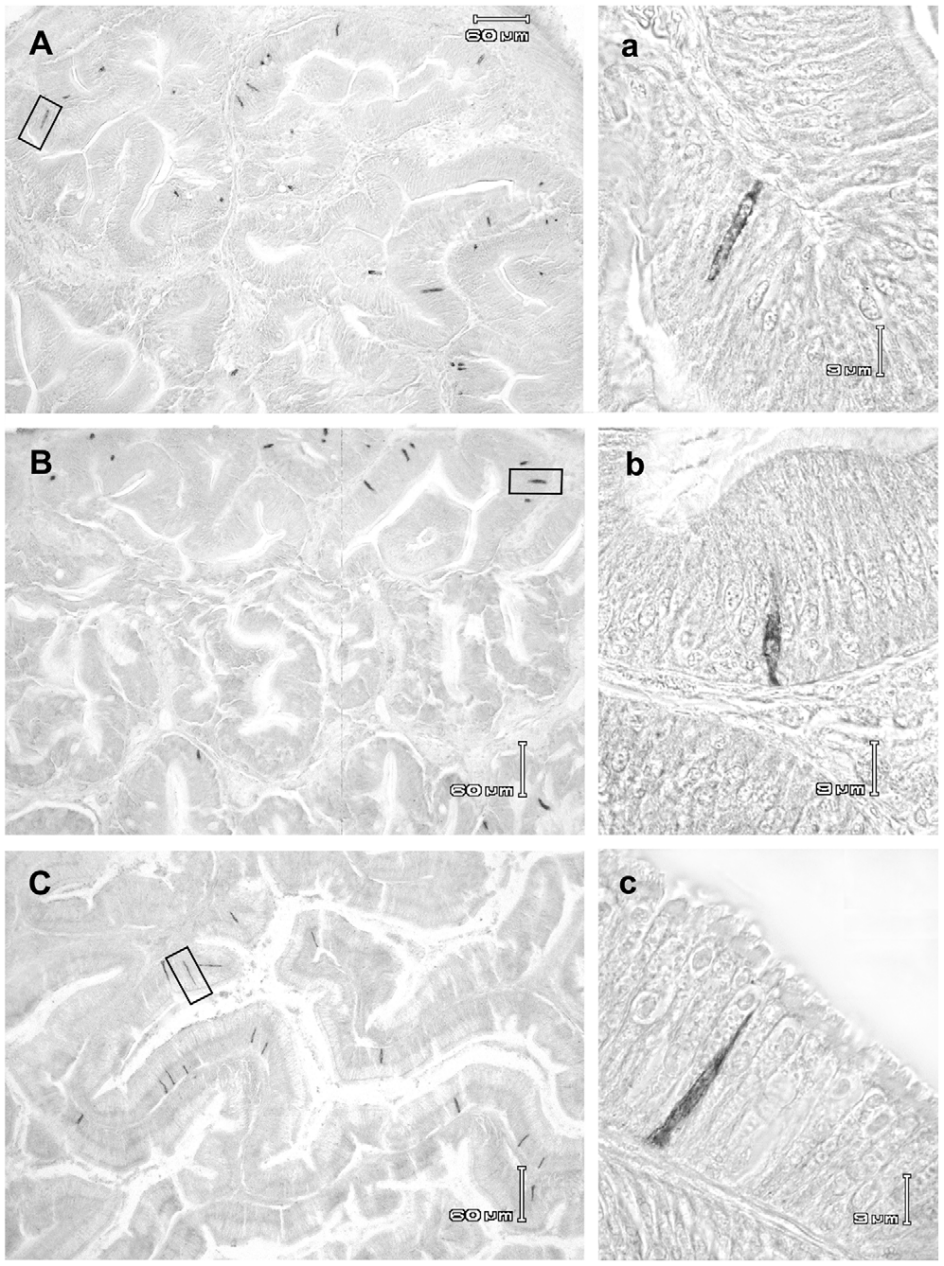
Distribution of CCK endocrine cells in the gut mucosa of *D. sargus*. The cells were immunoistochemically detected by reaction against anti-flounder CCK10 antibody. (A) Pyloric caeca mucosal folds from a fed specimen *D.sargus*. (B) Pyloric caeca mucosal folds from a fasted specimen *D.sargus*. (C) Hindgut mucosal folds from a fasted specimen *D.sargus*. (a), (b), and (c) are enlargements of cells boxed in (A), (B), and (C), respectively.

## Discussion

This study identified full-length nucleotide and amino acid sequences encoding two different isoforms of prepro-CCK in the white sea bream digestive tract. The deduced pre-prohormones were named CCK-1 and CCK-2, since they were shown to belong to the fish CCK-1 and CCK-2 subfamilies, respectively, by phylogenetic analysis. It has been suggested that the presence of multiple CCK isoforms in several fish species, as demonstrated in flounder, puffer, zebrafish, Atlantic salmon, rainbow trout, and white sea bream (present study) has probably been generated by an extra genome duplication event in teleosts compared to tetrapods [40]. On the basis of Bayesian tree evaluation, it can be suggested that both white sea bream CCKs followed a significant degree of diversification from diverse ancestors, while the length of their branch suggests that CCK-2 gene diversified more recently compared to CCK-1 gene.

Comparative alignment analyses of the obtained sequences demonstrated that the highest degree of similarity for white sea bream CCK-1 occurred with flounder CCK1 and yellowtail CCK (84%), followed by puffer CCK1 (76%). On the other hand, white sea bream CCK-2 showed the highest similarity with both flounder and puffer CCK2 (80%). The two isoforms were similar at 47.4%, and shared a common C-terminal octapeptide, DYLGWMDF, as flounder CCK1 and CCK2 [12]. The C-terminal octapeptide, which appears to be the major product of post-translational processing in fish [11], is extremely well conserved among species, with the only variable amino acid in position 6 from the C-terminus, which is leucine in both white sea bream isoforms, as well as the majority of teleosts, and methionine in tetrapods and elasmobranches. This conservation suggests that CCK has conserved biological functions among fish species. Unlike white sea bream, rainbow trout isoforms have leucine, asparagine or threonine in position 6 [11], spotted river puffer leucine or valine [12], and Atlantic salmon leucine or asparagine [13].

CCK-immunoreactivity was demonstrated in the present study in all intestinal segments of white sea bream, included the hindgut, as in turbot [25]. The CCK-10 antibody used in the present study, which had proven successful on the congener species *Diplodus puntazzo* [23], had been raised against Japanese flounder CCK1, whose terminal sequence DRDY**L**GW**M**DF is identical to those of both white sea bream CCK-1 and CCK-2. On the other hand, the possibility of a cross-reaction of antibody with gastrin could be excluded. In fact, unlike mammal gastrin and CCK, which share the C-terminal tetrapeptide sequence WMDF, white sea bream gastrin C-terminal octapeptide DY**Q**GW**V**DF (GenBank Accession No. JN210580) differs from CCKs in two amino acids, in positions 3 and 6, respectively. The localization of CCK-ir cells within the mucosal lamina propria, where vagal afferent peripheral terminals lie, agrees with the hypothesis that release of CCK from endocrine cells may activate vagal afferent terminals by a paracrine mode of action [41].

qPCR revealed that both CCK isoforms (i.e., CCK-1 and CCK-2) were expressed in the hindgut, as well as in the other intestinal segments. This is the first report of multiple CCK isoforms expressed throughout the whole intestine in the same individual of a given species. In previous studies, regional analysis of CCK expression demonstrated that rainbow trout CCK-N, unlike CCK-L and CCK-T isoforms, was expressed in pyloric caeca and anterior midgut, but not in posterior midgut and hindgut [11]. Similarly, salmon CCK-N, unlike ubiquitary CCK-L isoform, was found to be expressed only in pyloric caeca [13]. Such different pattern of CCK expression in salmonids, led Murashita et al. [13] to hypothesize a novel physiological role for CCK, alternative to control of digestive processes.

In order to ascertain whether white sea bream CCK may be implied in the feedback control of uncompleted digestive processes in the hindgut, CCK mRNA expression responses to feeding and fasting were analyzed for the first time in all intestinal segments, from pyloric caeca through the hindgut. A significant decreased pattern of CCK-2 mRNA expression in all intestinal tracts, including hindgut, was apparent after 72 h starvation in white sea bream. Decreased CCK levels in the most proximal tracts of intestine during fasting have been reported also in yellowtail [24], [42]. However, in the present study, decreased CCK-2 expression was also found in posterior midgut and hindgut, suggesting that CCK-2 in the distal tract of intestine may participate in the feedback control of digestion in white sea bream, even if at a minor extent, as suggested by a significantly lower expression, compared to the proximal tract. Similarly to CCK-2 mRNA expression,CCK protein expression decreased in fasted fish. However, it was not affected by fasting in the hindgut. On the other hand, white sea bream CCK-1 mRNA expression followed a completely different pattern. In fact, its expression abundance did not seem to be related to intestinal topography, as CCK-2. Moreover, CCK-1 expression was found to increase after 72 h starvation in all intestinal segments, except for the hindgut, where no significant difference was apparent between fed and fasted fish. Therefore, a different role from that of CCK-2 may be hypothesized for white sea bream CCK-1 isoform, based upon their different response to starvation, and, interestingly, such role does is not related to feeding or fasting in the hindgut.

To summarize the present study, two CCK isoforms were demonstrated in the hindgut of *D.sargus*, one of which (CCK-2) may be involved in the feedback control of uncompleted digestive processes. On the other hand, a functional role alternative to regulation of digestive processes may be inferred for *D.sargus* CCK-1, since its expression was unaffected by feeding or fasting.
